# The influence of experimental inflammation and axotomy on leucine enkephalin (leuENK) distribution in intramural nervous structures of the porcine descending colon

**DOI:** 10.1186/s12917-018-1496-y

**Published:** 2018-05-24

**Authors:** Slawomir Gonkowski, Krystyna Makowska, Jaroslaw Calka

**Affiliations:** Department of Clinical Physiology, Faculty of Veterinary Medicine, University of Warmia and Mazury, Oczapowski Str, 13 Olsztyn, Poland

**Keywords:** Leucine-enkephalin (leuENK), Enteric nervous system (ENS), Inflammation, Axotomy, Colon, Pig

## Abstract

**Background:**

The enteric nervous system (ENS), located in the intestinal wall and characterized by considerable independence from the central nervous system, consists of millions of cells. Enteric neurons control the majority of functions of the gastrointestinal tract using a wide range of substances, which are neuromediators and/or neuromodulators. One of them is leucine–enkephalin (leuENK), which belongs to the endogenous opioid family. It is known that opioids in the gastrointestinal tract have various functions, including visceral pain conduction, intestinal motility and secretion and immune processes, but many aspects of distribution and function of leuENK in the ENS, especially during pathological states, remain unknown.

**Results:**

During this experiment, the distribution of leuENK – like immunoreactive (leuENK-LI) nervous structures using the immunofluorescence technique were studied in the porcine colon in physiological conditions, during chemically-induced inflammation and after axotomy. The study included the circular muscle layer, myenteric (MP), outer submucous (OSP) and inner submucous plexus (ISP) and the mucosal layer. In control animals, the number of leuENK-LI neurons amounted to 4.86 ± 0.17%, 2.86 ± 0.28% and 1.07 ± 0.08% in the MP, OSP and ISP, respectively. Generally, both pathological stimuli caused an increase in the number of detected leuENK-LI cells, but the intensity of the observed changes depended on the factor studied and part of the ENS. The percentage of leuENK-LI perikarya amounted to 11.48 ± 0.96%, 8.71 ± 0.13% and 9.40 ± 0.76% during colitis, and 6.90 ± 0.52% 8.46 ± 12% and 4.48 ± 0.44% after axotomy in MP, OSP and ISP, respectively. Both processes also resulted in an increase in the number of leuENK-LI nerves in the circular muscle layer, whereas changes were less visible in the mucosa during inflammation and axotomy did not change the number of leuENK-LI mucosal fibers.

**Conclusions:**

LeuENK in the ENS takes part in intestinal regulatory processes not only in physiological conditions, but also under pathological factors. The observed changes are probably connected with the participation of leuENK in sensory and motor innervation and the neuroprotective effects of this substance. Differences in the number of leuENK-LI neurons during inflammation and after axotomy may suggest that the exact functions of leuENK probably depend on the type of pathological factor acting on the intestine.

## Background

**Fig. 1:**
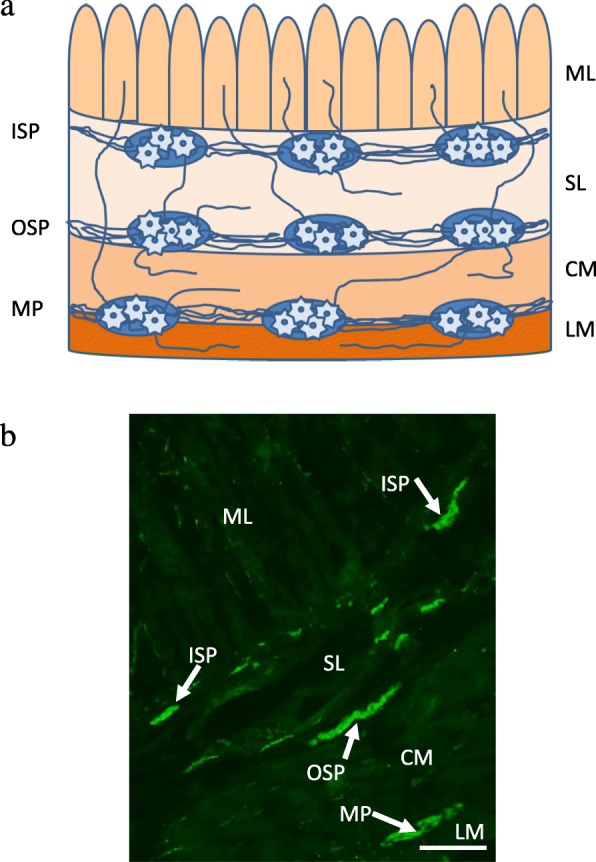
The organization of the enteric nervous system (ENS) in the porcine descending colon. **a** the scheme; **b** the view under florescent microscope, where nervous structures are labelled with directed towards protein gene product 9.5 (PGP 9.5) used as panneronal marker. LM – longitudinal muscle layer; CM – circular muscle layer; submucosal layer; ML – mucosal layer; MP – myenteric plexus; OSP – outer submucous plexus; ISP – inner submucous plexus. Bar 100 μm

It is well-known that neural control of all functions of the gastrointestinal (GI) tract is performed by both extrinsic innervation [1–4] and the enteric nervous system (ENS) [5, 6]. Localization of extrinsic neurons supplying the stomach and intestine depend on the innervated regionof the GI tract. These neurons may be located in prevertebral sympathetic ganglia, sympathetic chain, dorsal root ganglia, sensory and parasympathetic ganglia of vagal nerve, as well as in parasympathetic nuclei of sacral spinal cord [1, 2, 4, 6]. In turn, the ENS is situated in the wall of the GI tract. It is composed of a large number of neurons, which are characterized by considerable independence from the central nervous system and, for this reason, it has also been called the “second” or “intestinal” brain [7]. The structure of the ENS depends on the species studied and the regionof the GI tract. In porcine, large intestine enteric neurons are grouped into three separate ganglionated plexuses, which are interconnected with a dense network of nerves (Fig. 1). There are three types of these plexuses: myenteric plexus (MP) – located between the longitudinal and circular muscles, outer submucous plexus (OSP) – immediately adjacent to internal side of the circular muscle layer and inner submucous plexus (ISP) – positioned between muscularis mucosa and lamina propria [5, 8]. Neuronal cells within the above-mentioned plexuses belong to various functional classes, play different functions and show the presence of a broad range of active substances which, importantly, can play various roles as neuromediators and/or neuromodulators [6].

It should be pointed out that the ENS plays a key role in the regulation of intestinal functions not only in physiological conditions, but also during various pathological states. This fact may be expressed by structural, functional or chemical changes within enteric neurons, which may be a result of adaptive responses to operating factors. Till now, such changes in the ENS have been observed under physiological changes, such as development, aging or diet modification, but they are more visible during pathological processes, including intestinal and systemic diseases or nerve injury [8–11].

One of the many active substances occurring within enteric neurons is leucine-enkephalin (leuENK) which, along with methionine-enkephalin, is an endogenous opioid. These substances are pentapeptides derived from a precursor (proenkephalin) and described for the first time within the porcine brain in the 1970s [12]. Later investigations found that leuENK may also arise from α-neo-endorphin, dynorphin A or dynorphin B, which derived from prodynorphin [13]. Enkephalins, like as exogenous opioids (e.g. morphine) act via a different type of G-protein coupled receptors, including δ-opioid, κ-opioid and μ-opioid receptors [14, 15]. Although the main action of enkephalins is a mechanism connected with analgesia, previous studies have shown that these substances are also involved in other physiological processes, such as regulation of respiratory [16], urinary [17] or circulatory [18] systems.

To date, enkephalins have been described within the GI tract in various mammals species, including humans, in both the ENS and intestinal mucosal enterochromaffin cells [15, 19–21] as well as in the extrinsic innervation of intestine [1, 2]. Even so, the exact roles of opioids within the digestive system are not entirely clarified, although it is known that these substances mainly take part in reducing visceral pain [14]. Other functions of opioids within the GI tract include participation in intestinal immune processes, as well as inhibition of intestinal motility and secretion [14, 22, 23]. The inhibitory effects of enkephalins mentioned above cause an increase in food transit time in the GI tract and may contribute to constipation [14]. In addition, some previous studies have described changes in the number of enteric neurons immunoreactive to enkephalins under pathological factors, which suggests the participation of these substances in adaptive or neuroprotective processes within the intestine [1, 2, 21]. Nonetheless, many questions connected with the functions of leuENK in the ENS during pathological processes are not clear, especially in the GI tract of the pig, which is increasingly becoming an optimal laboratory animal due to well-established anatomical, biochemical and physiological resemblances to humans [24, 25]. These similarities primarily concern the organization of the ENS [26], which makes the pig a perfect animal model for experiments on changes in enteric neurons under various pathological factors.

The aim of the present investigation was to describe, for the first time, the influence of selected pathological factors, including experimental inflammation and axotomy on leucine enkephalin-like immunoreactive (leuENK-LI) enteric neurons in the porcine descending colon. It should be pointed out that the selection of this fragment of the GI tract as a subject of the present study was also not accidental, since various pathological processes often develop within the large intestine both in human and animals [8, 27–30].

## Methods

### Experimental animals

The present experiment was performed on 20 immature sows of the Large White Polish breed at the age of 8 weeks (about 18 kg body weight) bought on the commercial farm of pig production in Bałcyny (Poland). Animals were kept in normal laboratory conditions in the animal quarters of Faculty of Veterinary Medicine, University of Warmia and Mazury, Olsztyn (Poland) with feeding typical for this species and age of animal. The pigs were maintained in the pens (5 animals in each pen) with an area of about 4 m^2^. All experimental procedures, as well as number of experimental animals were consistent with the instructions and agreements of the Local Ethical Committee in Olsztyn (Poland), with special attention paid to minimizing any stress reaction during and after surgery (agreement numbers: 90/2007 from 20 November 2007 and 85/2008 from 17 December 2008). Animals during unloading after the transport were randomly (without any determined method) divided into four groups, each of which consisted of five pigs located in separate pens. Then the assignment of animals from each pen to the particular procedures were performed by drawing lots. Experimental groups were as follows: control (C group) – without any experimental procedures, control 1 (C1) – animals subjected to “sham” operations, inflammatory (I group) - pigs with chemically-induced colitis and axotomy (A group), where animals were subjected to the cutting of specific nerves (see below).

**Fig. 2:**
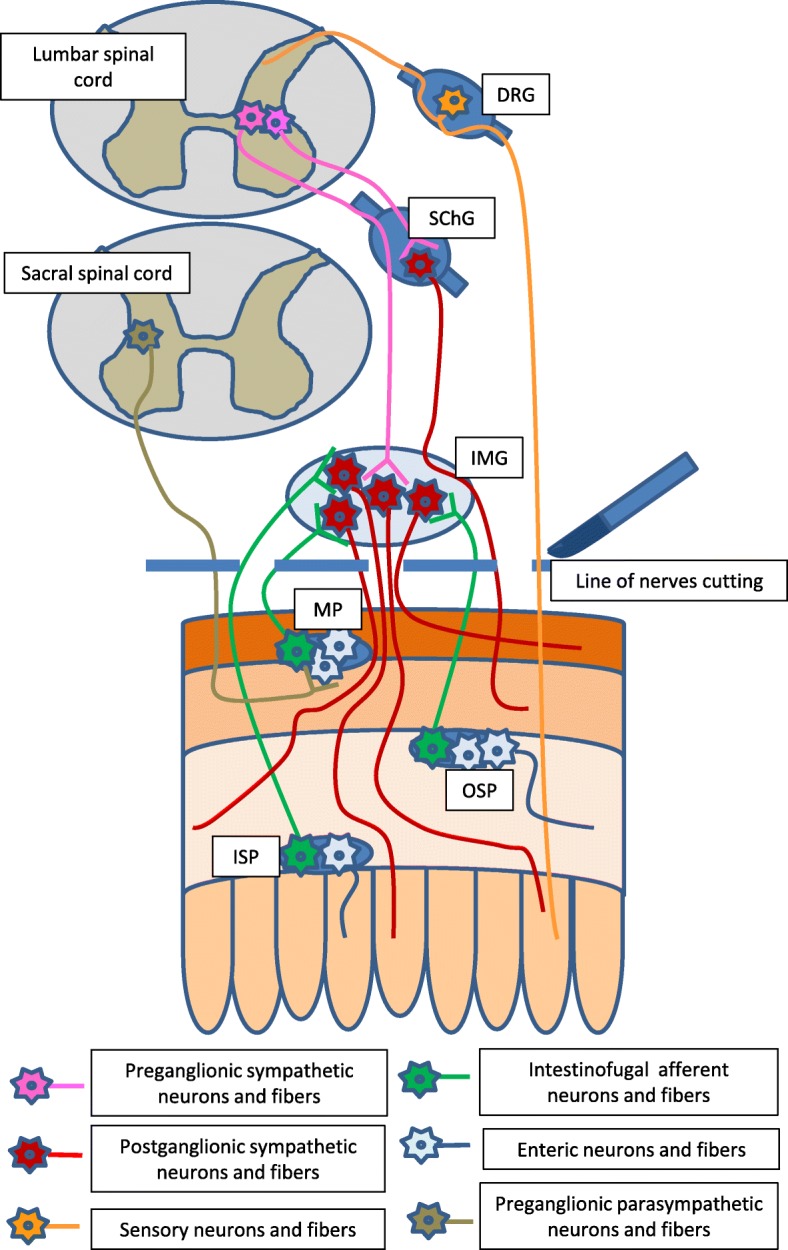
Different types of nerve fibers, which were interrupted during the cutting of caudal colonic nerves: DRG – dorsal root ganglion; SChG - sympathetic chain ganglion; IMG – inferior mesenteric ganglion; MP – myenteric ganglion; OSP – outer submucous ganglion; ISP – inner submucous ganglion

After five-day adaptive period the experiment was started. The sows from C1, I and A groups were subjected to median laparotomy performed under general anaesthesia, which consisted of premedication with Stressnil (Janssen, Belgium, 75 μl/kg of body weight, given intramuscularly) 15 min. Before the administration of the main aesthetic – sodium thiopental (Thiopental, Sandoz, Kundl-Rakúsko, Austria; 20 mg/kg of body weight, given intravenously) prior to the surgery. Chemically-induced inflammation and axotomy were made using the methods previously described by Gonkowski et al. [31]. According to these methods, the animals of group I were injected with 80 μl of 10% formalin solution in saline (microinjections of 5–8 μl) into the wall of the descending colon (to a depth of 0,2–0,5 mm) in an area, where nerves from the inferior mesenteric ganglia supply the gut. Surgery on animals of group A consisted of the bilateral transection of caudal colonic nerves connecting to the inferior mesenteric ganglion with the descending colon (Fig. 2). Animals of group C1 were subjected to “sham” operations, which were aimed at exclusion of surgical manipulations on the enteric nervous system. Pigs of group C1were injected in the same manner as group I, but a pure saline solution was used instead of formalin. After 5 days, all animals were anaesthetized in the same way as described above and euthanized by an overdose of sodium thiopental and immediately perfused transcardially with freshly prepared 4% buffered paraformaldehyde (pH 7.4).

### Double-labelling immunofluorescence technique

Segments of the descending colon (approximately 2 cm long) from the area where nerves from the inferior mesenteric ganglia supply the colon were collected from all pigs used in the experiment. Inflammation in the animals of group I was confirmed by a histopathological examination, which was made at the Laboratory of Histopathology (Faculty of Veterinary Medicine, University of Warmia and Mazury, Olsztyn, Poland). Immediately after collection, colonic fragments were post-fixed by immersion with 4% buffered paraformaldehyde (pH 7.4) for 20 min and rinsed in phosphate buffer (0.1 M, pH 7.4, at 4 °C) for 3 days with the exchange of buffer every day. The tissues were then put into 18% phosphate-buffered sucrose (at 4 °C) for 2 weeks. Finally, they were frozen at -22 °C. After freezing samples were coded and since then the assessors did know which samples belonged to which experimental group. Coding of samples was performed to avoid any bias in the evaluation of colonic fragments. Then tissues were cut perpendicular to the lumen of the colon into 14-μm-thick sections using microtome (Microm, HM 525, Walldorf, Germany) and fixed on glass slides.

The fragments of colon were subjected to standard double-labelling immunofluorescence, which has been described previously by Gonkowski et al. [31, 32]. During the present study, the combination of two kinds of primary antisera were used: mouse monoclonal antibody directed towards protein gene-product 9.5 (PGP 9.5, Biogenesis, UK, working dilution 1:2000, used here as a pan-neuronal marker) and rabbit polyclonal anti – leucine-enkephalin antibody (leuENK, Abcam, UK, working dilution 1:1000). The primary antisera mentioned above were visualized by a combination of species-specific secondary antibodies, i.e. Alexa fluor 488 donkey anti-mouse IgG and Alexa fluor 546 donkey anti-rabbit IgG (both from Invitrogen, Carlsbad, CA, USA, working dilution 1:1000).

Standard control probes were made to test the specificity of primary antibodies. These included pre-absorption, omission and replacement of primary antibodies by non–immune sera. The pre-absorption test consisted of incubation (for 18 h at 37 °C) of primary antibodies in working dilutions with the appropriate antigen. Antibodies directed towards PGP 9.5 were pre-incubated with native human protein gene product 9.5 (AbD Serotec, UK), and anti-leuENK antibody with synthetic human leucine-enkephalin peptide (Abcam, UK). The concentration of each antigen was 20 μg per 1 ml of diluted antibody. This procedure, as well as omission and replacement of primary antisera by non-immune sera, completely eliminated specific stainings.

### Counting the nerve structures and statistical analysis

To delimit the percentage of enteric neurons immunoreactive to leuENK, at least 600 PGP-9.5- positive cells in a particular enteric plexus (MP, OSP and ISP) of each studied animal were examined for leuENK-like immunoreactivity. Double-labelled perikarya (only neurons with clearly visible nucleus) were evaluated using an Olympus BX51 microscope equipped with epi-fluorescence and appropriate filter sets. The obtained results were pooled and presented as mean ± SEM. To prevent double counting of leuENK-LI neurons, the sections were located at least 100 μm apart.

For semi-quantitative evaluation of the density of leuENK-LI nerves within the enteric ganglia, an arbitrary scale was used, where (−) indicated the absence of studied fibers, (+) - single fibers, (++) - rare fibers, (+++) – dense network of fibers and (++++) depicted a very dense meshwork of fibers studied.

In turn, denotation of the density of intramuscular and intramucosal nerves immunoreactive to leuENK was based on counting all leuENK-LI nerve fibers *per* microscopic observation field (0.55 mm^2^). Nerves were counted in four sections per animal (in 5 fields per section) and the obtained data were pooled and presented as a mean. It should be pointed out that the nerves in the intestine can form very small bundles and it is not always possible to count exact numbers of fibers in fibre bundles. Therefore the present study includes only fibers that could have been counted.

All pictures were captured by a digital camera connected to a PC. Statistical analysis was carried out with an Anova test (Graphpad Prism v. 2.0; GraphPad Software Inc., San Diego, CA, USA). The differences were considered statistically significant at *p* ≤ 0.05. No statistical power calculation was conducted prior to the study, and the determination of the size of animal experimental groups was based on data available in the previous studies, where the number of five animals in the case of pigs during neuro-immunofluorescence investigations are commonly accepted.

## Results

During the present investigation, leuENK-positive nervous structures were observed within the descending colon in animals of all experimental groups and their number clearly depended on both the pathological factor studied and the part of the ENS (Table 1).

**Table 1:** Leucine-enkephalin – like immunoreactive nervous structures in the porcine descending colon

Bowel part		No. of animal	C Group	C1Group	I Group	A Group
CML^1^		1	16.40	14.60	30.80	23.30
		2	17.15	17.15	28.90	26.20
		3	13.80	12.85	32.60	24.15
		4	13.15	14.50	29.05	23.15
		5	16.00	14.40	32.75	26.80
		average	15.30 ± 0.77^a^	14.70 ± 0.69^a^	30.82 ± 0.83^b^	24.72 ± 0.75^c^
MP	CB^2^	1	5.06	5.13	9.25	6.12
		2	5.23	5.15	12.06	8.63
		3	4.53	3.80	9.92	6.10
		4	4.38	4.30	11.44	7.60
		5	5.10	5.62	14.73	6.05
		average	4.86 ± 0.17^a^	4,80 ± 0.33^a^	11,48 ± 0.96^b^	6,90 ± 0.52^c^
	NF^3^	average	++	++	++++	+++
OSP	CB^2^	1	1.90	1.58	8.70	8.26
		2	3.05	3.38	8.76	8.68
		3	2.80	3.58	9.15	8.46
		4	2.90	1.38	8.60	8.15
		5	3.64	3.93	8.35	8.75
		average	2.86 ± 0.28^a^	2.77 ± 0.53^a^	8.71 ± 0.13^b^	8.46±12^b^
	NF^3^	average	+++	+++	+	+
ISP	CB^2^	1	0.84	0.58	7.52	3.48
		2	1.27	1.02	11.94	5.92
		3	1.07	0.60	9.61	4.64
		4	1.20	0.86	8.20	3.60
		5	0.98	1.34	9.73	4.76
		average	1.07 ± 0.08^a^	0.88 ± 0.14^a^	9.40 ± 0.76^b^	4.48 ± 0.44^c^
	NF^3^	average	+	+	+	+
S/ML^1^		1	0.85	1.20	3.00	0.85
		2	1.30	1.00	4.45	1.30
		3	1.15	1.30	4.20	1.20
		4	1.45	2.00	2.80	1.15
		5	1.50	1.15	3.65	1.95
		average	1.25 ± 0.12^a^	1.33 ± 0.17^a^	3.62 ± 0.32^b^	1.29 ± 0.18^a^

*C group* control animals, *C1group* “sham” operated animals, *I group* pigs suffering from inflammation, *A group* animals after axotomy

*CML* circular muscle layer, *MP* myenteric plexus, *OSP* outer submucous plexus, *ISP* inner submucous plexus, *S/ML* submucosal/mucosal layer, *CB* cell bodies, *NF* nerve fibers

^1^Average number of nerve profiles per area studied (mean ± SEM)

^2^Relative frequency of particular neuronal subclasses is presented as % (mean ± SEM) of all neurons counted within the ganglia stained for PGP 9.5

^3^The density of intraganglionic nerve fibers positive for leuENK is presented in arbitrary units

Statistically significant data (*p* ≤ 0.05) between C group and group C1, I and A in the number of leuENK-LI nervous structures within particular part of the colon are marked by different letters and not significant data are marked by the same letters

**Fig. 3:**
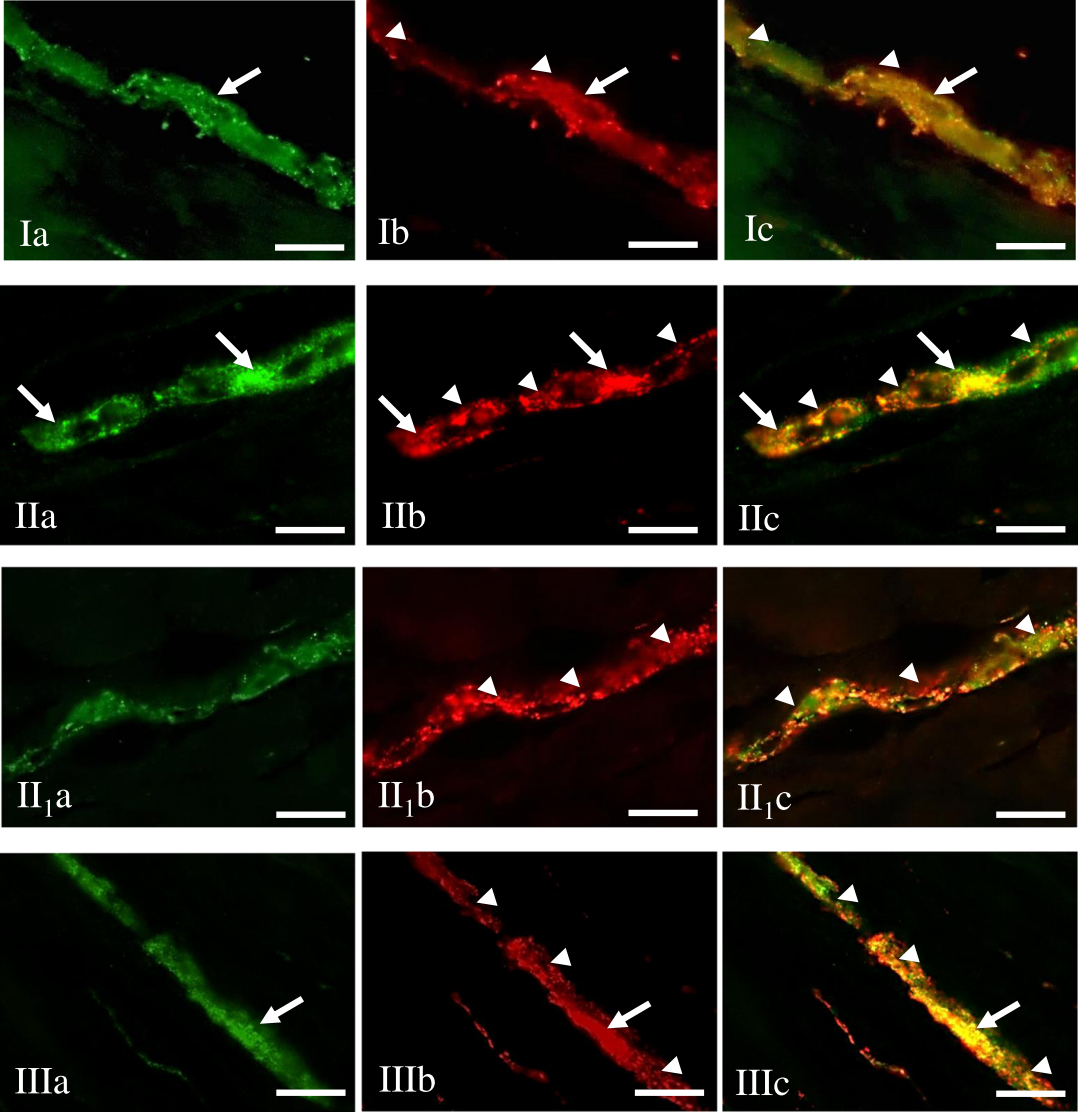
Myenteric plexus of the porcine descending colon under physiological conditions (I), during inflammation (II, II1) and after axotomy (III) immunostained for PGP 9.5 (a) and leuENK (b). The right column of the pictures (c) shows the overlap of both stainings. Co-localisation of both antigens is indicated with arrows (perikarya) and arrow heads (nerve fibers). Bar, 20 μm

**Fig. 4:**
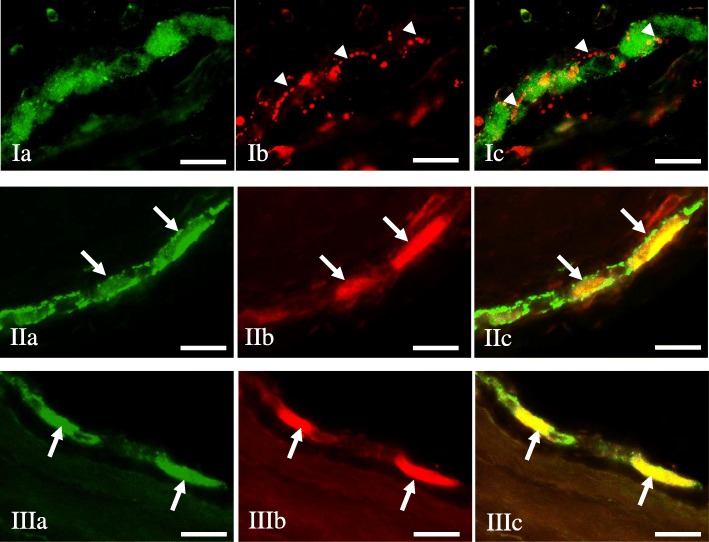
Outer submucous plexus of the porcine descending colon under physiological conditions (I), during inflammation (II) and after axotomy (III) immunostained for PGP 9.5 (a) and leuENK (b). The right column of the pictures (c) shows the overlap of both stainings. Co-localisation of both antigens is indicated with arrows (perikarya) and arrow heads (nerve fibers). Bar, 20 μm

Under physiological conditions, a relatively small number of leuENK-LI neurons was noted in all “kinds” of enteric plexuses (Table 1). The percentage of such neuronal cells (in relation to number of all PGP 9.5-LI neurons) amounted to 4.86 ± 0.17%, 2.86 ± 0.28% and 1.07 ± 0.08% in MP, OSP and ISP, respectively. Regarding the view of individual enteric ganglia, only single neuronal cells immunoreactive to leuENK were noted in control animals (Fig. 3. I), but the majority of ganglia did not show the presence of leuENK-LI neurons (Figs. 4.I and 5.I). Moreover, intraganglionic leuENK-positive nerve fibers were observed in all kinds of enteric ganglia. The dense network (+++) of these nerves were encountered in OSP, whereas in MP they were rare (++), and in ISP only single (+) nerve processes immunoreactive to leuENK were noted (Table 1). A relatively dense network of clearly visible LeuENK-positive nerves (Fig. 6.Ia) was also observed in the circular muscle layer (15.30 ± 0.77 nerves/observation field). In turn, only single, thin and delicate leuENK-LI nerves were noted in the mucosal layer (Fig. 6.Ib).

**Fig. 5:**
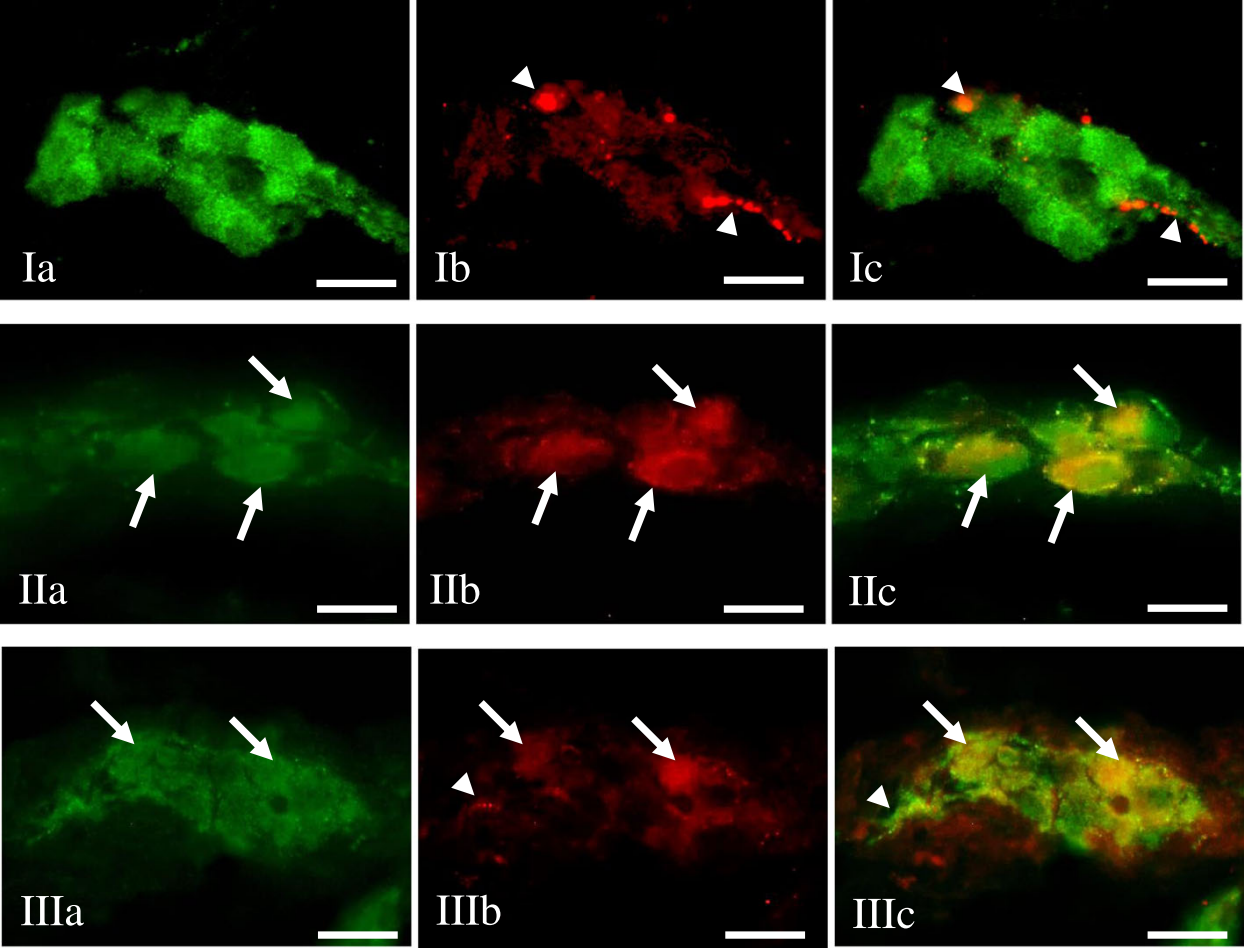
Inner submucous plexus of the porcine descending colon under physiological conditions (I), during inflammation (II) and after axotomy (III) immunostained for PGP 9.5 (a) and leuENK (b). The right column of the pictures (c) shows the overlap of both stainings. Co-localisation of both antigens is indicated with arrows (perikarya) and arrow heads (nerve fibers). Bar, 20 μm

**Fig. 6:**
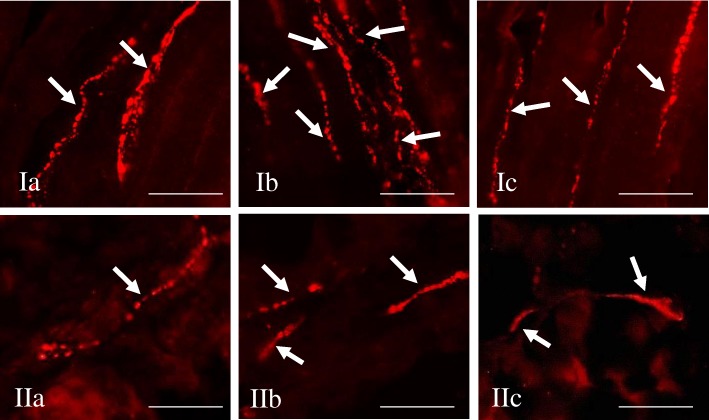
Distribution pattern of nerve fibers (arrows) immunostained for leuENK in the circular muscle layer (I) and mucosal layer (II) of porcine descending colon under physiological conditions (a), during inflammation (b) and after axotomy (c). Bar, 20 μm

Statistically significant differences were not observed between control and “sham” operated animals, but both pathological states studied changed the immunoreactivity to leuENK. These changes are generally expressed by an increase in the number of leuENK-positive neuronal structures, but their intensity depended on the pathological factor acting on the intestine (Table 1).

The highest percentage of neurons immunoreactive to leuENK were found within the myenteric plexus during the inflammatory process (Fig. 3.II), where the number of these cells increased by above 6.5 percentage points (pp). These changes were accompanied by an increase in the density of leuENK-LI intraganglionic nerve fibers. In animals suffering from inflammation, two “kinds” of myenteric ganglia were observed. One of them was characterized by the presence of leuENK-LI cells and a not very dense network of fibers immunoreactive to this peptide (Fig. 3.II). In the other MP, only very dense intraganglionic leuENK-LI nerves were visible, without leuENK-positive neurons (Fig. 3.II_1_). Axotomy also caused an increase in the number of leuENK-LI neurons and intraganglionic fibers in the MP (Fig. 3.III), but these changes were less visible.

The pathological states studied caused an increase in the percentage of leuENK- positive neuronal cells located in OSP (Fig. 4). Contrary to MP, the differences between inflammation and axotomy were not observed. Both investigated states produced an increase in the number of leuENK-LI neurons by about 5.5 pp. (Table 1). These changes in OSP were accompanied by a decrease in the density of intraganglionic nerve fibers immunoreactive to leuENK (Fig. 4).

An increase in the leuENK-positive neuron number was also noted in the ISP (Fig. 5). As with myenteric plexus, more visible modifications were observed during the inflammatory process, where the percentage of leuENK-LI cells grew nine-fold in relation to control animals and amounted to 9.40 ± 0.76% of all neurons immunoreactive to PGP 9.5. This was the clearest change of all parts of the ENS studied. Axotomy also caused an increase in the number of cells immunoreactive to leuENK, but changes were less visible (Table 1). Contrary to the number of leuENK-positive neurons, the density of leuENK-LI intraganglionic nerves in the ISP was not changed during the pathological factors studied (Table 1).

Both inflammation and axotomy caused an increase in the density of leuENK-positive nerve fibers in the circular muscle layer (Fig. 6.I). In this case, as in the MP and ISP, more visible changes were observed during inflammatory process, where the number of fibers immunoreactive to leuENK was doubled versus control animals (Table 1). In contrast, the number of leuENK-positive nerves localized in the mucosal layer only increased during inflammation, whereas axotomy did not change their number (Fig. 6.II, Table 1).

## Discussion

During the present investigation, leuENK-positive nerve structures have been observed in all parts of the ENS studied, as well as in intramuscular and intramucosal nerve fibers. These structures under physiological conditions (in spite of nerves within circular muscle layer) were rather limited. This is in agreement with the majority of previous studies, where the presence of leuENK has been described both in the ENS and extrinsic innervation of the GI tract in various mammal species, including humans [15, 19, 21]. The results obtained during the present study show that distribution of leuENK-positive nervous structures in the descending colon of the pig and other studied species are similar. In the pig (like in rats, guinea pigs or cats) such structures were first observed in the myenteric plexus and the circular muscle layer, whereas in the mucosa and submucosal enteric ganglia they are rather rare [20, 33, 34]. Particular similarities in the distribution of leuENK-positive nervous structures in the intestinal wall may also be observed between humans [15, 35, 36] and pigs (this study). This suggests that relatively well-known resemblances in the ENS organization of both these species [26] also apply to the distribution of leuENK.

Moreover, it should be pointed out that there are some discrepancies in the localization of porcine enteric neurons immunoreactive to leuENK in the light of previous studies. For example, in the stomach, some investigations have noted the absence of this peptide in the ENS [37], other authors have described relatively numerous leuENK-LI neurons, especially in the MP [38]. The reason for these discrepancies remains unclear, although they strongly suggest that enteric neurons can change leuENK expression under various physiological factors, such as nutritional differences, farming factors and/or environmental bacterial flora, which in all probability is associated with some currently unknown functions of opioids within the ENS.

Till now, opioids (including leuENK) in the digestive tract have been described as factors affecting the nociception, intestinal immune functions, motility and secretion [14] and their exact roles depends on both the type of opioid and receptors class. Opioid receptors, belonging to G-protein coupled receptors, have been described in the central and peripheral nervous structures, including the enteric nervous system [39–41] and three kinds of them (δ-opioid, κ-opioid and μ-opioid receptors) can participate in the regulation of gastrointestinal physiology. Previous studies showed that each class of opioid receptors has its preferred ligand and regulates specific processes. κ-opioid receptors, which bind mainly with dynorphin and μ-opioid receptors (their ligand is β-endorphin), are primarily responsible for visceral antinociception [14, 41]. In turn, δ-opioid receptors, preferred ligands of which are enkephalins, first of all exhibit an inhibitory effect on the intestinal motility, causing an increase in the transit time of digestive tract content [14]. Moreover, previous studies have described other gastrointestinal functions of opioids, such as suppression of intestinal secretion, participation in immune processes and protective roles during inflammation [14].

During the present study, both chemically-induced inflammation and axotomy caused changes in the number of leuENK-positive colonic nerve structures, and the influence of inflammatory processes has been more visible. The differences in leuENK-like immunoreactivity have probably been connected with roles of opioids in intestinal immune functions. It is known that these substances, as well as their receptors, are present in macrophages, monocytes and some classes of lymphocytes and take part in mechanisms connected with cytokines production and T-cells proliferation [14, 42]. Another reason for the observed changes may be the participation of enkephalins in sensory stimuli conduction and anti-nociceptive mechanisms, which has been described within both the central nervous system and the enteric neurons [43–45].

Moreover, it is well-established that opioids can reduce the activity of intestinal muscles by various mechanisms, such as a reduction in the release of stimulating neurotransmitters, changes in the excitability of enteric neurons and/or neuromodulatory effects on intestinal motor neurons [14, 41, 46]. Enkephalins, acting through δ-opioid receptors, which are located mainly in the myenteric plexus of the ENS, are also a major factor reducing intestinal motility [14]. It is possible that changes observed during inflammation, where an often excessive increase of muscular contractility and diarrhoea are present, may result from compensatory activity of enteric neurons. In this case, the increase of leuENK expression would have to be aimed at peristalsis inhibition and return to physiological conditions. This assumption is all the more probable that the most numerous leuENK-LI nervous structures have been noted during the inflammatory process in the muscular plexus and circular muscle layer. Since enkephalins are also known as inhibitors of intestinal secretions, the mechanisms of changes noted in the ISP could be similar to those mentioned above.

Changes in leuENK-like immunoreactivity observed in animals suffering from inflammation may also result from the participation of opioids in mechanisms connected with typical symptoms of gastrointestinal diseases, including nausea and emesis [14] and/or from neuroprotective actions of these substances. Previous studies have shown that synthetic agonists of δ-opioid receptors may prolong the cell life of many organs, such as heart, lung or kidney [47, 48], as well as have a neuroprotective influence on neuronal cells in various regions of the brain [49–51] and spinal cord [52]. It is not yet known whether endogenous or synthetic enkephalins can prolong the life of neurons supplying the GI tract in the pig, but the results obtained during the present study, as well as previous investigations on extrinsic innervation of porcine digestive tract [2, 53, 54], where pathological factors also caused an increase in leuENK-like immunoreactivity strongly suggest this fact, because it is well-established that injury to neurons causes an increase in the expression of active substances which promote the regeneration of injured cells [55].

During the present study, changes in the number of leuENK-LI nervous structures after axotomy have been less visible than during inflammation. The cutting of nerves connecting the inferior mesenteric ganglia with the descending colon caused the destruction of various types of nerves (Fig. 2). The majority of these were postganglionic sympathetic nerves, axons of neurons located predominantly in the inferior mesenteric ganglia as well as in the sympathetic chain. Other nerves derived from afferent sensory cells located in dorsal root ganglia, parasympathetic preganglionic neurons in sacral spinal cord or intestinofugal afferent (also named “viscerofugal”) neurons (IFANs). IFANs, belonging to the ENS, are situated in the wall of intestine, in all kinds of enteric plexuses, send axons to prevertebral sympathetic ganglia, including coeliac ganglion, cranial mesenteric ganglion or inferior mesenteric ganglion (depending on the segment of the GI tract) and take part in intestino – intestinal reflexes without the participation of the central nervous system [56–58]. Thus, changes in leuENK-like immunoreactivity noted after axotomy may result from compensative activity of enteric neurons after the destruction of colonic extrinsic innervation or neuroprotective processes in IFANs, which is more likely in light of the relatively well-known neuroprotective effects of enkephalins, which were discussed above.

It should be pointed out that the mechanisms of changes observed during the present investigation may be variable. They can arise from an increase in leuENK synthesis in enteric neurons or modifications in intraneuronal transport of this substance from the cell body to nerve endings. In the case of fluctuations in leuENK synthesis, the changes may be due to different types of changes during transcription, translation or post-translational processes.

## Conclusions

The present study shows that leuENK-positive nervous structures are present in the porcine descending colon and their distribution is similar to other species, especially to humans, which suggests the possibility of using the pig as a model animal in investigations of this peptide in the human ENS. Moreover, leuENK-like immunoreactivity in the porcine ENS undergoes fluctuations during chemically-induced inflammation and after axotomy. The increase in the number of leuENK-LI neurons and nerves can suggest neuroprotective roles of the described substance within the ENS. The present results are the first step toward exploitation of synthetic enkephalins as a medicine, not only during colitis, which has been suggested by previous studies [45], but also after intestinal innervation damage. On the other hand, the observed changes clearly depended on both the part of the ENS and the type of factor studied. This is probably caused by different roles of leuENK in intestinal innervation, according to the type of acting pathological stimulus. These roles are still not fully explained and require further study.

## Abbreviations

A group: Animals after axotomy
C group: Control group
C1 group: “Sham” operated animals
ENS: Enteric nervous system
GI tract: Gastrointestinal tract
I group: Inflammatory group
ISP: Inner submucous plexus
leuENK: Leucine enkephalin
MP: Myenteric plexus
OSP: Outer submucous plexus
Pp: Percentage point
SEM: Standard error of the mean
